# Editorial: Separation and analytical chemistry

**DOI:** 10.3389/fchem.2023.1298452

**Published:** 2023-11-02

**Authors:** Pankaj Kumar Parhi

**Affiliations:** Department of Chemistry, Fakir Mohan (FM) University, Balasore, Odisha, India

**Keywords:** ionic liquid, liquid liquid extraction, green chemistry, metal, extraction

Secondary solid wastes, encompassing e-waste, battery waste, spent catalysts, magnet waste, and scrap alloys, are critical sources of bearing considerable quantity of valuable metals such as Cu, Co, Ni, Mo, V, W; rare-earth elements (REEs) Nd, Sm, Sc, Pr, and Dy; and precious metals Ag and Au in this century (Behera et al., 2020; Jyothi et al., 2020; Parhi and Misra, 2022). For this reason, researchers have significantly shifted their attention to developing sustainable technology for the treatment of these secondary wastes to recover highly pure metals vis-à-vis disposal of zero waste to the environment (Jyothi et al., 2020). Of the two extractive metallurgical approaches, namely, pyro-metallurgy and hydrometallurgy, the latter has been preferentially chosen as a suitable processing method over the former because of issues of high energy consumption and emissions (Behera et al., 2020). In the hydrometallurgical approach, the most common methods are chemical leaching in the upstream stage and solvent extraction in the downstream stage, which are employed for extraction and separation processes, and for the purification of desired metals from the secondary phases (Parhi and Misra, 2022). The use of mineral acid solvent reagents such as HCl, H_2_SO_4_, and HNO_3_ during conventional leaching, and traditional solvent reagents such as organo-phosphorus derivatives (D2EHPA, PC88A, Cyanex 272, TOPO, TBP), chelating types (LIX series), and secondary/tertiary amines (Alamine 336, Alamine 304, Alamine 308) in the solvent extraction process, are very common (Jyothi et al., 2020; Le and Lee, 2020). These solvents are applied because of their reasonable efficacy and limited selectivity towards the extraction of a metal of interest from an aqueous medium depending on the nature and existence of metals in the respective secondary waste or corresponding leach liquor phases.

The majority of investigations (Jyothi et al., 2020; Le and Lee, 2020) reveal that the existence of metals as an oxide phase in secondary wastes appears to be highly prone to becoming leached with solvent, namely, HCl, while metals found in sulfide or alloy form and/or other mixed category in the secondary waste phases are readily subjected to treatment with H_2_SO_4_ and HNO_3_ in order to dissolve the metal content. Sometimes pre-treatment operations, including roasting, mechanical activation such as ball milling, baking, microwave (MW) treatment, and ultra-sonication (UW), are performed in order to activate or oxidize/reduce the waste phases for attaining a leachable phase for substantial metal extraction with high efficacy in subsequent leaching stages (Behera et al., 2019). However, a paradigm shift has been noticeable over the course of time in hydrometallurgy to the comprehensive adoption of several organic solvent reagents such as acetic acid, citric acid, oxalic acid, and tartaric acid as alternatives to mineral acids for the leaching of metals from various secondary wastes (Mohanty et al., 2021; Mohanty et al., 2023). These acidic solvents are not only found as potential reagents for the leaching of metals from the aforementioned secondary wastes but are also considered green solvents, as they do not have any environmental emission issues and have a regeneration ability. Unlike conventional leaching, advanced pre-treatment approaches such as MW and UW can also be employed during organic-mediated leaching, mainly in the cases of low leaching behavior observed in direct organic acid leaching. The investigations on the extraction of Nd and Sm from magnet waste and Cu, Cr, and V from spent catalysts using acetic acid and citric acid reported these methods to be effective and promising. Enriched extraction with high efficacy was observed in the leaching of metals with the adoption of mechanical activation such as ultra-sonication along with organic acid-mediated leaching (Behera et al., 2019).

Similar to the upstream stage operation, in the downstream solvent extraction process, green ionic liquids (ILs) have received considerable attention for their high rate of extraction ability, quick regenerative non-degradable nature, and wide temperature range of operation. ILs are molten salts comprising organic cations and organic/inorganic anions. The most common, imidazole and pyridinium-based solvent derivatives, are used extensively as suitable alternatives to traditional solvents (Yudaev and Chistyakov, 2022). In recent developments, bi-functional ionic liquids CYPHOS 101, CYPHOS 104, R_4_ND, R_4_NPC, and R_4_NCY can be synthesized from the organo-phosphorous-based solvents in combination with quaternary ammonium chloride. These solvents are used in the extraction of REEs, heavy metals, and strategic metals as a replacement for traditional solvents in solvent extraction processes (Sola et al., 2020; Mohapatra et al., 2022). The bi-functional ionic liquid is prepared either by combining traditional organo-phosphorous derivatives with quaternary ammonium/quaternary phosphonium chloride or modifying the functional group, for example, replacing the chloride of quaternary salt with nitrate or thiocyanate. R_4_NSCN, R_4_PSCN, TOMAC, TOMAN, CYPHOS 101, CYPHOS 104, R_4_ND, R_4_NPC, and R_4_NCY certainly show great potential, not only in the context of their high extraction ability in the loading of the base, transition, and rare earth metals, but they also exhibit high selectivity during the extraction of metals in the presence of others from numerous secondary waste leach liquor phases (Jyothi et al., 2020; Parhi and Misra, 2022). The future scope of the application of green ionic liquid solvents in extraction systems is not restricted to only the liquid–liquid extraction process but also utilization in various extraction/separation technologies. The key salient features of these green IL reagents, such as low solvent inventory, greater loading ability with a low dose supplement, and usages in pure form, assure promising performances for further use in other separation methods including supported liquid membrane (SLM), emulsion liquid membrane (ELM), and solvent impregnated resin (SIR) systems.

In general, ILs at room temperature are often highly viscous, and many ILs are moisture-sensitive and highly hygroscopic in nature. The major drawback encountered in the use of ILs in their pure form exclusively in industrial-scale extraction operations is their highly viscous nature. This may lead to slow mass transfers, difficulties while pumping and stirring, and very low solid–liquid separation during extraction (Asrami et al., 2021). Furthermore, the recyclability or regeneration of ILs both in batch and industrial scale operations is another issue that needs to be addressed in order to adopt a continuous mode of operation in hydrometallurgical solvent extraction processes. One way to overcome the issues of viscosity and recyclability of ionic liquids is to combine the solvent extraction circuit and the electrolysis circuit during extraction and recover the desired metals from numerous secondary and other waste phases in which the resulting metal-loaded organic solvent from the solvent extraction circuit can be directly fed into the electrolytic cell as an electrolyte to regenerate ILs vis-à-vis obtaining highly pure metals. However, some ILs, including imidazole and pyridine derivatives, are expensive solvent reagents, and although they are used extensively for the extraction of heavy metals and REEs in the laboratory, the scope of application in industrial extraction processes is limited. This can be overcome by synthesizing tailor-made quaternary ammonium/phosphonium derivative bi-functional ILs (R_4_ND, R_4_NCY, R_4_PCY, R_4_PSCN, etc.) (Parhi et al., 2023). However, further research studies are necessary for the successful adoption of ILs in industrial hydrometallurgical extraction processes.
